# Contamination of Hospital Inanimate Surfaces with Methicillin-Resistant *Staphylococcus aureus* and Extended-Spectrum Beta-Lactamase producing bacteria at Ocean Road Cancer Institute, Dar es Salaam, Tanzania

**DOI:** 10.1371/journal.pone.0352189

**Published:** 2026-06-23

**Authors:** Donath Mkenda Valerian, Mtebe Majigo, Ninael Jonas, Loveness Urio, Emanuel Magembe, Reuben Abednego, Modest Benard, Doreen Kallanga, Ibrahim Mauki, Agricola Joachim

**Affiliations:** 1 Department of Epidemiology and Biostatistics, Muhimbili University of Health and Allied Sciences, Dar es Salaam, Tanzania; 2 Tanzania Field Epidemiology and Laboratory Training Program, Dar es Salaam, Tanzania; 3 Njombe Regional Referral Hospital, Njombe, Tanzania; 4 Department of Microbiology and Immunology, Muhimbili University of Health and Allied Sciences, Dar es Salaam, Tanzania; 5 National Public Health Laboratory, Dar es Salaam, Tanzania; Hawassa University College of Medicine and Health Sciences, ETHIOPIA

## Abstract

**Background:**

Methicillin-resistant Staphylococcus aureus (MRSA) and extended-spectrum beta-lactamase (ESBL) producing bacteria are the most common contaminants on hospital surfaces. Hospitalized patients, especially those with cancer, have a higher likelihood of acquiring hospital-associated infections in a contaminated environment. Outbreaks of MRSA and ESBL-producing bacteria linked to contaminated inanimate hospital surfaces are a well-documented threat. Cancer patients, who often have prolonged hospital stays, are particularly at risk of acquiring these multidrug-resistant (MDR) infections. The immunosuppressed state of cancer patients further increases their susceptibility, resulting in higher morbidity and mortality associated with these infections. This study assessed the prevalence, antimicrobial resistance, and factors associated with contamination of inanimate hospital surfaces by MRSA and ESBL-producing bacteria.

**Materials and methods:**

This was cross-sectional study conducted at Ocean Road Cancer Institute, Dar es salaam, Tanzania in March and April 2023. The surfaces were conveniently selected, and a total of 247 inanimate surface samples were collected using a sterile swab pre-moistened with sterile Trypticase soy broth. A structured data collection checklist was used to record key variables for each sampled surface. The samples were cultured on Mannitol salt agar and MacConkey agar containing 2 mg/ml of ceftazidime. Identification of the isolates were done by using VITEC MS. Methicillin-resistant Staphylococcus aureus (MRSA) and extended-spectrum beta-lactamase (ESBL) producing bacteria were confirmed phenotypically through testing with a 30 µg cefoxitin disk and the combination disk method, respectively. Antimicrobial susceptibility testing was performed using the Kirby-Bauer disk diffusion method. Logistic and modified Poisson regression analyses were performed to determine factors associated with contamination.

**Results:**

A total of 247 swab samples were collected from six predetermined items; bed rails, computer keyboards, door handles, hand-washing sinks, nursing station tables, and trolleys. The proportions of MRSA and ESBL producing bacteria’ contamination were 21 (8.5%) and 63 (25.5%), respectively. *Acinetobacter baumannii* and *Klebsiella pneumoniae* were the predominant ESBL producing bacteria each accounting for 38 of the 84 isolates (45.2%). All *Staphylococcus aureus* isolates were identified as MRSA and were non-susceptible to at least one antimicrobial agent in three or more antibiotic classes; however, all isolates showed 100% susceptibility to linezolid. *Klebsiella pneumoniae* demonstrated resistance to piperacillin-tazobactam, cefepime, and ciprofloxacin, with rates ranging from 92.1% to 97.4%. Resistance to meropenem was observed in 52.6% of *Klebsiella pneumoniae* isolates. Hand-washing sinks were the only surface independently associated with contamination by ESBL-producing bacteria (APR 5.8, P < 0.001).

**Conclusion:**

A significant proportion of inanimate hospital surfaces were contaminated with MRSA and ESBL-producing bacteria. The findings imply a need to improve infection prevention and control practices.

## Introduction

Contamination of inanimate hospital surfaces remains a significant concern, posing a considerable risk of Health-care-associated infections (HAIs) to hospitalized patients particularly those who are immunocompromised, such as cancer patients [1,2]. Methicillin-resistant *Staphylococcus aureus* (MRSA) and extended-spectrum beta-lactamase (ESBL) producing bacteria are multidrug resistant (MDR) pathogens frequently implicated in HAIs, contributing to long hospital stay, raise in morbidity and mortality [3,4]. The burden is disproportionately higher in low- and middle-income countries such as those in Africa, Asia, the Caribbean, and Latin America [5].

Inanimate surface contamination by MRSA and ESBL-producing bacteria has been documented globally. In France, contamination rates have reached up to 91% for premises near patients and 88% farther away [6]. MRSA contamination on inanimate surfaces in Moroccan hospitals has been reported at 45% [7]. Extended-spectrum beta-lactamase (ESBL) producing bacteria contamination rates in high-income countries like England are lower at approximately 3.4% [8], while in African countries like Tanzania, rates reach up to 32.5% [9]. These data highlight the widespread presence of multidrug-resistant organisms on hospital surfaces and the potential for pathogen transmission. In addition to high prevalence, outbreaks of MRSA and ESBL-producing bacteria linked to contaminated hospital surfaces have been widely reported, highlighting their potential to cause rapid transmission among hospitalized patients [10,11]

Cancer patients are exceptionally endangered because prolonged hospitalization and immunosuppression increase their risk of acquiring HAIs [12]. A reported HAI rate of 40.7% among cancer patients underscores this issue [13]. A significant factor in HAIs transmission is the presence of inanimate surfaces [2,10]. MRSA and ESBL-producing bacteria have been isolated from these surfaces [7,9]. These bacteria circulate among patients and persist on inanimate surfaces [14]. In Tanzania previous studies in tertiary hospitals have documented substantial contamination of inanimate surfaces with MRSA and ESBL-producing bacteria [15,16]. The colonization of patients and healthcare workers by ESBL-producing bacteria has been shown to correlate with the contamination of inanimate surfaces [17]. Therefore, it is crucial to identify the types of bacteria contaminating the hospital’s inanimate surfaces and their antibiotic resistance patterns in specialized cancer treatment centers, where patients are highly immunocompromised, and the inanimate surface contamination data are lacking.

Infection prevention and control (IPC) measures are essential in the fight against HAIs [18]. Standard precautions ensure removal and reduction of contamination of inanimate surfaces by MRSA and ESBL-producing bacteria [19]. Non-adherence to IPC contributes to the spread and persistence of these bacteria on surfaces [20]. Monitoring the hospital environment, particularly inanimate surfaces, is critical for HAI prevention and control [21]. Surfaces that harbor pathogens create sites for multiplication, necessitating more stringent decontamination to ensure a safe patient care environment [22,23].

Unpublished data from this setting have shown the presence of bacterial infections in neutropenic cancer patients. Contaminated inanimate surfaces were hypothesized to be a potential source of infection. Therefore, this study determined the proportion, antimicrobial resistance patterns, and factors associated with MRSA and ESBL-producing bacterial contamination at the Ocean Road Cancer Institute (ORCI), Dar es salaam, Tanzania.

## Materials and methods

### Study design and setting

This was a cross-sectional study conducted at the Ocean Road Cancer Institute (ORCI) Dar es salaam, Tanzania, the largest cancer care facility in Tanzania in March and April 2023. The hospital serves cancer patients nationwide and private patients from foreign countries. With 270 beds, it serves about 50,000 patients annually. The institute also runs outreach programs, assisting around 15,000 clients across Tanzania. ORCI is a teaching hospital for Muhimbili University of Health and Allied Sciences (MUHAS) located in Dar es salaam, Tanzania [24].

### Study population

The study population comprised frequently touched inanimate surfaces in the hospital.

### Inclusion and exclusion criteria

The study included inanimate surfaces in clinical areas and excluded surfaces in non-clinical areas, such as administration offices

### Sample size estimation

The minimum sample size (n) was calculated using Kish and Leslie formula


$$\begin{array}{c} \frac{\text{n}={\text{Z}}^{\text{2}}\;\times \text{P}(\text{1}-\text{P})}{\varepsilon^{\text{2}}} \end{array}$$

where

n = Sample size

Z = Standard normal deviate set at 1.96 for 95% confidence level

P = Estimated proportion of MRSA and ESBL-producing bacteria hospital inanimate surface contamination, set at 0.201 from [15].

ε = Margin of error set at 5% (0.05)

Therefore,


$$\begin{array}{c} \frac{\text{n }=\;\text{1}.\text{96 }\times \;\text{1}.\text{96 }\times \;\text{0}.\text{201 }(\text{1}-\text{0}.\text{201})\;=\text{ 247}}{\text{0}.\text{05 }\times \;\text{0}.\text{05}} \end{array}$$

The number of samples collected from each ward was obtained based on Probability proportional to size (PPS) of items present in the respective wards.

### Sampling methods and sample collection

Samples were collected from predetermined inanimate surfaces that were accessible and available. These surfaces were conveniently selected and included door handles, bed rails, nursing station tables, trolleys, computers, and hand-washing sinks. Each sample was taken using a sterile swab pre-moistened with sterile Trypticase soy broth once daily, one hour after routine cleaning. The swab was rolled over 25 cm² from left to right at a right angle. Each swab was kept in trypticase soy broth, and all swabs collected that day were transported in a cooler box to the National Public Health Laboratory for processing.

### Data collection tool

A structured data collection checklist was used to record key variables for each sampled surface. The variables included were the source of the sample (type of surface), ward location, sex of room occupants, number of patients per room, availability of hand-washing stations, and surface characteristics, including porosity and smoothness. Ward location was included as floor levels (first floor for VIP patients; second and third floors for regular patients). The rationale for including ward location was to enable comparison of contamination levels between VIP and general wards, consistent with the hospital’s ward naming. Porous surfaces included water-permeable materials such as wooden surfaces (e.g., nursing station tables). Non-porous surfaces included metal items such as trolleys, bed rails, and door handles. Rough surfaces included wooden tables, whereas smooth surfaces included door handles, bed rails, and trolleys. These variables were selected based on their potential influence on environmental contamination and HAIs transmission.

### Data quality assurance

Data collection and sample collection were conducted by the corresponding author, who is a trained laboratory professional familiar with microbiological sampling procedures. A structured data collection checklist was used to record all variables, and the tool was pretested in a similar hospital environment prior to data collection to ensure it captures all required information.

## Laboratory processing

### Isolation and identification of bacteria

This study focused specifically on MRSA and ESBL-producing bacteria due to their clinical significance as major MDR pathogens implicated in hospital-acquired infections and outbreaks, particularly among immunocompromised cancer patients and employed selective culture methods. The samples in trypticase soy broth were incubated at 35 ± 2 °C for 6 hours after being vortexed for 2 minutes. Subsequently, the samples were inoculated onto mannitol salt agar (Oxoid, UK) to isolate *Staphylococcus aureus* and onto MacConkey agar (Oxoid, UK) with and without 2 mg/ml of ceftazidime to screen for ESBL-producing bacteria. MacConkey agar without ceftazidime served as an internal control, to verify bacterial viability and media performance. Therefore, the absence of growth on ceftazidime-supplemented agar was interpreted as inhibition of non-ESBL-producing bacteria rather than failure of the culture process. Isolates from MacConkey agar with ceftazidime and those from mannitol salt agar with colony morphology resembling *Staphylococcus* spp. were sub-cultured on blood agar to obtain fresh colonies. Identification of bacteria to the species level was performed using VITEK MS, which employs the principle of Matrix-Assisted Laser Desorption Ionization Time-of-Flight (MALDI-TOF).

### Detection of ESBL-producing bacteria

The growth of suspected ESBL-producing bacteria on MacConkey agar (Oxoid, UK) supplemented with 2 mg/mL ceftazidime was confirmed using the combination disk method [21]. Briefly, a bacterial suspension matching the 0.5 McFarland turbidity standard was inoculated onto Mueller-Hinton agar plates. Subsequently, ceftazidime (30 µg) and cefotaxime (30 µg) discs, both with and without clavulanic acid (30 µg), were placed on the agar immediately. The plates were then incubated aerobically at 35°C ± 2°C for 18 hours. An increase in the diameter of the zone of inhibition of ≥5 mm for either ceftazidime-clavulanic acid or cefotaxime-clavulanic acid compared to ceftazidime and cefotaxime alone was interpreted as ESBL positive [25]. Standard reference strains of *E. coli* (ATCC 25,922) and *Klebsiella pneumoniae* (ATCC 700,603) were used for ESBL quality control.

### Detection of MRSA

MRSA was confirmed phenotypically using a 30 µg cefoxitin disk. A bacterial suspension with turbidity matching the 0.5 McFarland standard was inoculated onto Mueller-Hinton agar plates. Cefoxitin disks (30 µg) were then placed immediately on the agar. The plates were subsequently incubated under aerobic conditions at a controlled temperature of 35 ± 2°C for 18 hours. A zone of inhibition of ≤ 21 mm was interpreted as methicillin-resistant *Staphylococcus aureus* [25]. ATCC *25923 and* MRSA ATCC 43300 served as the negative and positive controls for MRSA, respectively.

### Antibiotic susceptibility testing

Antibiotic susceptibility testing was performed using the Kirby-Bauer disk diffusion method, following Clinical and Laboratory Standards Institute guidelines, 32nd edition (2022) [23]. MRSA isolates were assessed against gentamicin 10 µg, ciprofloxacin 5 µg, clindamycin 2 µg, erythromycin 15 µg, linezolid 30 µg, and trimethoprim-sulfamethoxazole 1.25/23.75 µg. For Gram-negative ESBL-producing bacteria, the antibiotics used were amikacin 30 µg, meropenem 10 µg, cefepime 30 µg, ciprofloxacin 5 µg, trimethoprim-sulfamethoxazole 1.25/23.75 µg, gentamicin 10 µg, aztreonam 30 µg, piperacillin-tazobactam 100/10 µg, and amoxicillin-clavulanic acid 20/10 µg. These antibiotics were selected based on Clinical and Laboratory Standards Institute (CLSI) recommendations and their common use in the treatment of infections at Ocean Road Cancer Institute. Internal quality control used [25]. MRSA ATCC 43300 and *Klebsiella pneumoniae* ATCC 700603 to verify disk and agar performance. The D-test was performed to detect inducible clindamycin resistance among clindamycin-sensitive isolates. Multi-drug resistance (MDR) was defined as non-susceptibility to at least one agent in three or more antimicrobial categories [26].

### Data analysis

Data analysis was conducted using STATA version 15. Descriptive statistics summarized the data, presenting categorical variables as frequencies and proportions. Bivariate and multivariable logistic regression identified factors associated with MRSA contamination. Modified Poisson regression assessed factors related to ESBL-producing bacteria contamination. Odds ratios for logistic regression and prevalence ratios for Modified Poisson, with 95% confidence intervals (CIs), were reported. Variables with p-value ≤ 0.2 in bivariate analysis were included in multivariable regression. Reference categories were selected based on epidemiological relevance and baseline comparison. Sex and number of patients were included as potential confounders despite p-values > 0.2. A correlation matrix assessed multicollinearity. Variables with p < 0.05 were considered statistically significant.

### Ethical approval

Ethical approval was obtained from the MUHAS Institutional Review Board (MUHAS-REC-11-2022-1448). Permission was granted by the Director General of Ocean Road Cancer Institute. No human participants were directly involved

## Results

### Sample distribution

A total of 247 surface swab samples were collected from six predetermined items. Most samples were taken from bed rails (89/247, 36.0%). The majority of samples were collected from rooms occupied by female patients (200/247, 81.0%). Seven-tenth of samples (71.6%;177/247) were collected from rooms with five or fewer patients. Furthermore, 89.1%, 220/247) of the samples were taken from premises with hand-washing stations. Most surfaces were non-porous (208/247, 84.2%) and smooth (228/247, 92.3%) (Table 1).

**Table 1:** Distribution of samples collected from the hospital inanimate surfaces.

Variable	Options	Frequency	Percent
**Source of the sample**	Bed rail	89	36.0
	Computer keyboard	23	9.3
	Door handle	37	15.0
	Hand-washing sink	49	19.8
	Nursing station table	28	11.3
	Trolley	21	8.5
**Ward location**	First floor	41	16.6
	Second floor	102	41.3
	Third floor	104	42.1
**Sex of room occupants**	Female	200	81.0
	Male	47	19.0
**Number of patients per room**	≤ 5	177	71.6
	6-10	70	28.3
**Hand-washing station availability**	No	27	10.9
	Yes	220	89.1
**Porosity of the surface**	Non-porous	208	84.2
	Porous	39	15.8
**Smoothness of the surface**	Rough	19	7.7
	Smooth	228	92.3

### Contamination of hospital inanimate surfaces with MRSA and ESBL-producing bacteria

**Fig 1:**
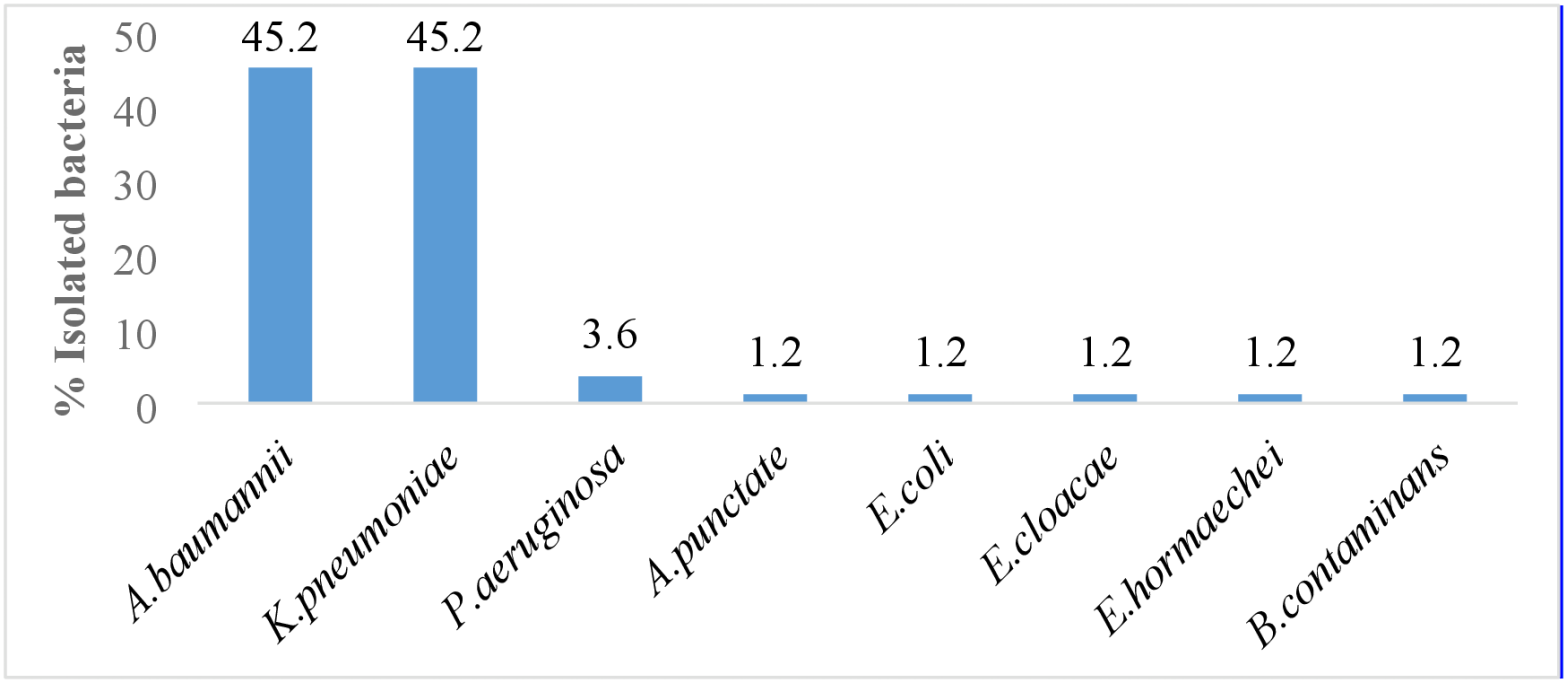
Distribution of ESB-producing bacteria isolated from hospital inanimate surfaces.

A total of 105 bacterial isolates were identified, 21(20%) MRSA and 84(80%) ESBL-producing bacteria. Of the 247 surface samples, 21 (8.5%) yielded *Staphylococcus aureus,* all of which were confirmed as MRSA. ESBL-producing bacteria were confirmed in 63 (25.5%) samples. Because some samples had multiple bacterial growths a total of 84 ESBL-producing bacteria were recovered from these 63 samples. *Acinetobacter baumannii* and *Klebsiella pneumoniae* were the predominant ESBL-producing bacteria, each accounting for 38(45.2%) of the 84 isolates. Other ESBL-producing bacteria isolated from the hospital inanimate surfaces are presented in Fig 1.

### Proportion of MRSA and ESBL-producing bacteria contamination

MRSA contamination was significantly higher in items from rooms occupied by male patients (9 out of 47, 19.2%) compared to rooms occupied by female patients (12 out of 200, 6.0%) (p = 0.004). Significant differences in ESBL-producing bacteria contamination were found to be related to the types of items sampled, the availability of hand-washing stations, and the porosity of the sampled surfaces. Hand-washing sinks showed the highest contamination rate, with ESBL-producing bacteria (39 out of 49 (79.6%) compared to other items (p < 0.001). Samples from premises with hand-washing stations had a higher rate of contamination with ESBL-producing bacteria (61 out of 220, 27.7%) than those from premises without hand-washing stations (2 out of 27, 7.4%) (p = 0.022). Furthermore, non-porous items exhibited the highest contamination rate with ESBL-producing bacteria (62/208, 29.8%), compared to porous surfaces (1/39, 2.6%) (p < 0.001) (Table 2).

**Table 2:** Comparison of MRSA and ESBL-producing bacteria contamination in hospital inanimate surfaces.

Variable		Total sample	MRSA contamination		ESBL contamination	
			Positive n (%)	P value	Positive n (%)	P value
**Overall**		247	21(8.5)		63(25.5)	
**Sampled Items**	Bed rail	89	12(13.5)	0.408	10(11.2)	<0.001
	Computer keyboard	23	1(4.4)		2(8.7)	
	Door handle	37	2(5.4)		5(13.5)	
	Hand-washing sink	49	3(6.1)		39(79.6)	
	Nursing station table	28	1(3.6)		5(17.9)	
	Trolley	21	2(9.5)		2(9.5)	
**Ward location**	First floor	41	4 (9.8)	0.069	11(26.83)	0.181
	Second floor	102	13 (12.8)		20 (19.6)	
	Third floor	104	4 (3.9)		32 (30.8)	
**Sex of room occupants**	Female	200	12 (6.0)	0.004	51(25.5)	0.996
	Male	47	9 (19.2)		12 (25.5)	
**Patients per room**	≤ 5	177	10(7.6)	0.603	43(24.3)	0.480
	6-10	70	11(9.5)		20(28.6)	
**Hand-washing station availability**	No	27	2(7.4)	0.829	2(7.4)	0.022
	Yes	220	19 (8.6)		61(27.7)	
**Porosity of the surface**	Non porous	208	19 (9.1)	0.410	62(29.8)	<0.001
	Porous	39	2 (5.1)		1(2.6)	
**Smoothness of the surface**	Rough	19	0 (0.0)		1(5.3)	0.35
	Smooth	228	21(9.2)		62(27.2)	

### Distribution of MRSA and ESBL-producing bacterial isolates by source of sample

*Acinetobacter baumannii* and *Klebsiella pneumoniae* were mainly isolated from hand-washing sinks, accounting for 50.0% and 84.2% of their respective isolates. Majotiy of the MRSA were obtained from bed rails (12/21, 57.1%). Other bacterial species and their distribution across sample sources are presented in Table 3.

**Table 3:** Distribution of MRSA and ESBL-Producing Bacterial Isolates by Source of Sample.

	Source of the sample						
ESBL-producing bacteria	Bed rail	Computer keyboard	Door handle	Hand washing sink	Nursing station table	Trolley	Total
*Acinetobacter baumannii*	9(23.7)	1(2.63)	5(13.16)	19(50.0)	3(7.9)	1(2.63)	**38**
*Aeromonas punctata*	0(0.0)	0(0.0)	0(0.0)	1(100.0)	0(0.0)	0(0.0)	**1**
*Burkholderia contaminans*	0(0.0)	1(100.0)	0(0.0)	0(0.0)	0(0.0)	0(0.0)	**1**
*Enterobacter cloacae*	0(0.0)	0(0.0)	1(100.0)	0(0.0)	0(0.0)	0(0.0)	**1**
*Enterobacter hormaechei*	0(0.0)	0(0.0)	0(0.0)	0(0.0)	0(0.0)	1(100.0)	**1**
*Escherichia coli*	0(0.0)	0(0.0)	0(0.0)	1(100.0)	0(0.0)	0(0.0)	**1**
*Klebsiella pneumoniae*	3(7.9)	0(0.0)	0(0.0)	32(84.2)	2(5.3)	1(2.6)	**38**
*Pseudomonas aeruginosa*	0(0.0)	0(0.0)	0(0.0)	3(100.0)	0(0.0)	0(0.0)	**3**
**Total**	**12(14.3)**	**2(2.4)**	**6(7.1)**	**56(66.7)**	**5(6.0)**	**3(3.6)**	**84**
MRSA	12(57.1)	1(4.8)	2(9.5)	3(14.3)	1(4.8)	2(9.5)	**21**

### Antibiotic resistance pattern of MRSA and ESBL-producing bacteria

We assessed the proportion of antimicrobial resistance in MRSA and ESBL-producing bacteria with more than 20 isolates. High resistance rates of MRSA were observed for clindamycin (81.0%), gentamicin (81.0%), and erythromycin (90.5%). Inducible clindamycin resistance was identified in 2 out of the 4 (50.0%) MRSA. All MRSA isolates were sensitive to linezolid.

Notably, *Klebsiella pneumoniae* showed a high rate of carbapenem resistance, with 52.6% of isolates resistant to meropenem. In addition, high resistance was observed to piperacillin–tazobactam (97.4%), cefepime (92.1%), ciprofloxacin (92.1%), aztreonam (89.5%), and trimethoprim–sulfamethoxazole (73.7%). *Acinetobacter baumannii* exhibited moderate resistance (21.1% − 52.6%) to most antibiotics tested, and was 100% susceptible to meropenem (Table 4).

**Table 4:** Antimicrobial resistance pattern of MRSA and ESBL-producing bacteria from hospital inanimate surfaces.

Bacteria	Antimicrobial resistance pattern (%)										
	MEM	TZP	ATM	FEP	CPR	CN	AK	SXT	E	LZD	CD
*Acinetobacter baumannii* n = 38	0.0	52.6	NA	23.7	31.6	23.7	21.1	44.7	NA	NA	NA
*Klebsiella pneumoniae* n = 38	52.6	97.4	89.5	92.1	92.1	18.4	18.4	73.7	NA	NA	NA
MRSA n = 21	NA	NA	NA	NA	71.4	81.0	NA	76.2	90.5	0.0	81.0

MEM- meropenem, TZP -piperacillin-tazobactam, ATM -aztreonam, FEP- cefepime, CPR- ciprofloxacin, CN- gentamicin, AK- amikacin, SXT- trimethoprim-sulfamethoxazole. AZM- azithromycin, CD- clindamycin, E- erythromycin, LZD- linezolid.

### Multidrug-resistant strains among ESBL-producing bacteria and MRSA isolates

Out of 38 *Klebsiella pneumoniae* isolates, 37 (97.4%) were identified as MDR, with 10 (26.3%) resistant to four or more classes. In comparison, only 17 (44.7%) of the *Acinetobacter baumannii* isolates were classified as MDR. Among the MRSA isolates, 13 out of 21 (61.9%) showed resistance to all five tested antibiotic classes. Notably, all MRSA strains were categorized as MDR (Table 5).

**Table 5:** Distribution of multidrug resistance (MDR) among MRSA and ESBL-producing bacterial isolates from hospital inanimate surfaces.

	MDR classes n (%)					
Bacteria	R3	R4	R5	R6	R7	MDR
*Acinetobacter baumannii* n = 38	7 (18.4)	5 (13.2)	4(10.5)	1(2.6)	0(0.0)	17(44.7)
*Klebsiella pneumoniae* n = 38	2 (5.3)	4 (10.5)	7(18.4)	16(42.1)	8(25.1)	37(97.4)
*Staphylococcus aureus n = 21*	6(28.6)	2(9.5)	13(61.9)	0(0.0)	0(0.0)	21(100.0)

MDR – multi-drug resistance, R3–R7 number of antimicrobial classes

### Factors associated with MRSA hospital inanimate surface contamination

In the bivariate logistic regression analysis, rooms occupied by male patients exhibited 3.7 times greater odds of MRSA contamination than those occupied by female patients (COR = 3.7; 95% CI: 1.5–9.4; p = 0.006). However, after adjusting for ward location and the number of patients per room, male room occupancy was not independently associated with MRSA contamination on hospital inanimate surfaces (AOR = 2.6; 95% CI: 0.9–7.2; p = 0.073) (Table 6).

**Table 6:** Bivariate and multivariable logistic regression analysis of factors associated with MRSA.

Variable	Total	MRSA	Bivariate analysis			Multivariate analysis		
		n(%)	COR	95%CI	P value	AOR	95%CI	P value
**Source of the sample**								
Door handle	37	2(5.4)	1					
Bed rail	89	12(13.5)	2.7	0.6-12.8	0.204			
Computer keyboard	23	1(4.4)	0.8	0.1-9.3	0.855			
Hand-washing sink	49	3(6.1)	1.1	0.2-7.2	0.888			
Nursing station table	28	1(3.6)	0.6	0.1-7.5	0.729			
Trolley	21	2(9.5)	1.8	0.2-14.1	0.557			
**Ward location**								
First floor	41	4(9.8)	1			1		
Second floor	102	13(12.8)	1.4	0.4-4.4	0.618	1.3	0.4-4.6	0.639
Third floor	104	4(3.9)	0.4	0.1-1.6	0.175	0.6	0.1-2.7	0.479
**Sex of room occupants**								
Female	200	12(6.0)	1			1		
Male	47	9(19.2)	3.7	1.5-9.4	**0.006**	2.7	0.7-6.6	0.132
**Number of patients per room**								
≤5	131	10(10.28)	1			1		
6-10	116	11(4.3)	0.4	0.1-1.4	0.148	0.6	0.2-2.3	0.440
**Hand-washing station availability**								
No	27	2 (7.4)	0.8	0.2-3.9	0.829			
Yes	220	19(8.6)	1					
**Porosity of the surface**								
Non porous	208	19 (9.1)	1					
Porous	39	2(5.1)	0.5	0.1-2.4	0.417			

COR- Crude Odd Ratio, CI- Confidence Interval, AOR- Adjusted Odds Ratio, MRSA – Methicillin-resistant *Staphylococcus aureus*.

### Factors associated with ESBL-producing bacteria hospital inanimate surface contamination

In modified Poisson regression analyses, only hand-washing sinks were found to be statistically significantly associated with contamination by ESBL-producing bacteria. In the bivariate Modified Poisson regression analysis, the prevalence ratio of ESBL-producing bacteria contamination in hand-washing sinks was 5.9 times higher compared to door handles (PR = 5.9; 95% CI: 2.6–13.5; *p* < 0.001). Hand-washing sinks had a 5.8 times higher prevalence ratio of ESBL-producing bacteria contamination compared to door handles after adjusting for other factors in multivariable modified Poisson regression analysis (PR = 5.8; 95% CI: 2.5–13.4; *p* < 0.001) (Table 7).

**Table 7:** Bivariate and multivariate modified Poisson regression analysis of factors associated with ESBL-producing bacteria Hospital inanimate surface contamination.

Variable	Total	ESBL producing bacteria	Bivariate analysis			Multivariate analysis		
			PR	95%CI	P value	APR	95%CI	P value
**Source of the sample**								
Door handle	37	5(13.5)	1			1		
Bed rail	89	10(11.2)	0.8	0.3-2.2	0.719	0.8	0.3-2.1	0.692
Computer keyboard	23	2(8.7)	0.6	0.1-3.1	0.579	0.7	0.1-4.9	0.740
Hand-washing sink	49	39(79.6)	5.9	2.6-13.5	<0.001	5.8	2.5-13.4	**<0.001**
Nursing station table	28	5(17.9)	1.3	0.4-4.1	0.632	2.8	0.9-8.6	0.081
Trolley	21	2(9.5)	0.7	0.1-3.3	0.659	0.7	0.1-3.4	0.639
**Ward**								
First floor	41	11(26.83)	1			1		
Second floor	102	20(19.6)	0.7	0.4-1.4	0.561	0.9	0.6-1.7	0.935
Third floor	104	32(30.8)	1.1	0.6-2.1	0.576	1.3	0.8-2.1	0.252
Sex of room occupants*								
Female	200	51(25.5)	1			1		
Male	47	12(25.5)	1.0	0.5-1.7	0.996	1.0	0.6-1.7	0.911
**Number of patients per room**								
≤5	131	43(24.3)	1			1		
6-10	116	20(28.6)	1.2	0.7-1.9	0.481	1.0	0.7-1.6	0.859
**Hand-washing station availability**								
No	27	2 (7.4)	0.3	0.1-1.0	0.056	0.9	0.2-4.3	0.924
Yes	220	61 (27.7)	1			1		
**Smoothness of the surface**								
Rough	19	1(5.3)	0.2	0.0-1.3	0.094	0.2	0.0-1.1	0.063
Smooth	228	62(27.2)	1			1		

PR- Crude Prevalence Ratio; APR – Adjusted Prevalence Ratio; CI – Confidence Interval; ESBL- Extended-spectrum beta-lactamases

## Discussion

The present study demonstrates that the environment around patients, particularly inanimate surfaces, can harbor medically significant pathogens such as MRSA and ESBL-producing bacteria. This is the first study conducted in a cancer treatment center in our area to assess contamination rates of hospital inanimate surfaces. The prevalence of MRSA contamination was (8.5%). Data on the contamination of hospital inanimate surfaces in cancer patient facilities are limited; therefore, comparisons were made with findings from general hospital environments. The MRSA prevalence observed in this study is comparable to reports from general hospital in South Africa and Michigan, USA [27,28], but lower than rates reported at MNH (19.5%) and tertiary hospital in Nepal (54.4% and 36%) [16,29]. Substantially a lower prevalence of 1.25% has been reported in Algeria [30].

A higher contamination rate may be attributed to overcrowding; some rooms at MNH accommodated more than ten patients, while the maximum in this study was ten. Variations in IPC practices could explain the differences. Bed rails were the most contaminated item for MRSA. Increased bed rail contamination was also reported at MNH by Nkuwi et al. [16]. A study in South Africa has documented high MRSA contamination in bed rails [27], probably because bed rails are frequently touched by colonized patients [31]. Rooms occupied by male patients showed the highest MRSA contamination, contrary to Nkuwi et al.’s findings, which reported higher contamination in female-occupied rooms [14]. Variations in hygiene practices might explain this. Regular disinfection is vital for removing pathogens from hospital surfaces [23].

ESBL-producing bacteria on inanimate surfaces significantly increase HAIs transmission [32]. This study found a high proportion (25.5%) of ESBL-producing bacteria, aligning with findings from a non-cancer hospital in Dar es salaam, Tanzania (32.5%) and Ethiopia (24.6%) [9,33]. A lower rate was noted in Ethiopia (14.8%) [34]. Differences in IPC practices may explain the disparities [22]. A high level of ESBL contamination was found in hand-washing sinks (79.6%). Similar results were reported in pediatric wards at MNH in Tanzania [9] and at a university hospital in Ethiopia [34]. This may be attributed to the sinks’ location within the toilet corridor, where they are used by admitted patients. Additionally, the same sinks are used for washing dishes, potentially introducing organic material that promotes bacterial growth [35]. The use of hospital sinks for purposes other than hand-washing has been linked to increased contamination by ESBL-producing Enterobacteriaceae [36,37]. The high contamination in sinks alerts patients and healthcare providers to the risk of nosocomial infection transmission.

*Klebsiella pneumoniae* and *Acinetobacter baumannii* were the most common ESBL-producing bacteria, making up 90%. These findings are consistent with two studies conducted in our settings [9,15]. Other research reported higher contamination rates with *Acinetobacter baumannii* and *Pseudomonas aeruginosa* [7,38]. The high proportion of ESBL-producing *Klebsiella pneumoniae* and *Acinetobacter baumannii* presents a significant infection risk for hospitalized cancer patients [13,39]. The WHO classifies these organisms as a critical priority due to their high resistance and clinical importance. They are included among WHO priority pathogens, emphasizing the need for targeted prevention and control [21]. Patients with dermatological cancer are at increased risk of infection from these bacteria, considered opportunistic pathogens in immunocompromised individuals, with notable resistance in the ESKAPE group [40].

The present study revealed high antimicrobial resistance to erythromycin, clindamycin, trimethoprim-sulfamethoxazole, and ciprofloxacin, increasing the risk of nosocomial infections in cancer patients. Similar findings were reported in studies from Nepal between 2018 and 2021 [29]. This resistance may have been accelerated by transmission of resistance genes from environmental strains colonizing inanimate surfaces [4]. No resistance was observed to linezolid. Similar observations were reported in Asian studies [4,41]. This may be due to limited prescription and availability of linezolid in our settings, preventing resistance development.

Notably, 77.3% of the isolates were MDR. A lower proportion was found in a study by Joachim et al. at MNH [9], possibly due to their focus on gram-negative bacteria, while this study included *Staphylococcus aureus*. Another study at MNH observed MDR in bloodstream infections at 70.5%, with 37% mortality, underscoring the risk of death linked to MDR nosocomial infections [42]. In the environment, MDR strains pose a risk of inadequate treatment for cancer patients, as MDR bacteria are difficult to treat. This also affects cancer patient survival rates [43].

*Klebsiella pneumoniae* and *Acinetobacter baumannii* were highly resistant to, piperacillin-tazobactam, aztreonam, cefepime, ciprofloxacin, and trimethoprim-sulfamethoxazole. *Klebsiella pneumoniae* showed resistance to meropenem, which indicates the presence of carbapenems-resistance strains circulating within the hospital, this finding suggests that cancer patients may be predisposed to infection with highly resistant organisms with significantly limited therapeutic options. underscoring the need for continuous surveillance and strengthened preventive measures within the hospital settings. Resistance to meropenem further limits treatment options for infections caused by ESBL-producing bacteria as it is typically reserved for multidrug-resistant infections. All *Acinetobacter baumannii* isolates showed 100% sensitivity to meropenem. Both strains showed low resistance to gentamicin and amikacin (23.7% and 21.1%). *Klebsiella pneumoniae* also showed low resistance to amikacin and gentamicin (18.4% each). These findings align with reports from studies in Tanzania and Ethiopia [33,44].

None of the analyzed factors were independently associated with MRSA. Although male occupancy showed an association in bivariate analysis, this relationship was not retained after adjustment, suggesting potential confounding. Similar inconsistencies have been reported in other studies [16]. For ESBL contamination, hand-washing sinks were significantly associated with contamination of hospital surfaces. Similar associations have been reported in the MNH settings [9]. Fecal contamination of sinks by users from toilets might explain the association, as studies have shown colonization with ESBL-producing bacteria shed through fecal material.

When interpreting the results of this study, it is important to consider that adherence to disinfection protocols and the efficacy of disinfectant was not assessed, which may have influenced the reported contamination rates. Equipment and air were not studied, and colony-forming units (CFUs) were not calculated; therefore, the findings cannot be directly compared with established standards used to inform infection control practices and assess the effectiveness of cleaning and disinfection protocols. Also, the use of nonprobability convenience sampling may limit the generalizability of the findings. Additionally, colonization of patients and staff with MRSA and ESBL-producing bacteria was not examined, nor were bacterial infections in admitted patients studied. This limits the ability to establish a direct link between the contamination of hospital inanimate surfaces and patient infections. Furthermore, we used phenotypic methods to detect MRSA and ESBL. These methods cannot definitively confirm the presence of mecA or specific ESBL genes. However, all recovered isolates were preserved and stored in a laboratory repository for future molecular characterization studies

## Conclusion

The study revealed that a significant number of inanimate hospital surfaces were contaminated with MRSA and ESBL-producing bacteria. Bed rails and hand-washing sinks emerged as the most contaminated sites. The presence of high levels of multidrug-resistant strains increases the risk of hospital-acquired infections, especially among immunocompromised cancer patients. Improving infection prevention and control measures is essential to reduce morbidity in this vulnerable population.

## Supporting information

S1 Data collection checklist. (DOCX)

S2 Anonymized data for factor analysis. (XLSX)

S3 Anonymzed data for ESBL Producers AST. (XLSX)

S4 Anonymized data for MRSA AST. (XLSX)
