# Supplementary material for: Contamination of Hospital Inanimate Surfaces with Methicillin-Resistant Staphylococcus aureus and Extended-Spectrum Beta-Lactamase producing bacteria at Ocean Road Cancer Institute, Dar es Salaam, Tanzania
Source: PLoS One. 2026 Jun 23;21(6):e0352189. doi: 10.1371/journal.pone.0352189 (PMC13289878; doi:10.1371/journal.pone.0352189)
Supplement: S1 — (DOCX) [file pone.0352189.s001.docx]

**DATA COLLECTION CHECKLIST**

**Sample ID Number:** _______________________________
**Date of Sample Collection:** ________________________

1. **Ward location:** __________________________________
2. **Room Number:** __________________________________
3. **Total Number of Patients in the Room:** _____________
4. **Sex of room occupants**
   ☐ Male
   ☐ Female
5. **Source of the sample**
   ☐ Door Handle
   ☐ Bed Rail
   ☐ Nursing Station Table
   ☐ Computer Keyboard
   ☐ Computer Mouse
   ☐ Hand-washing Sink
   ☐ Trolley
6. **Nature of the Surface 1**
   ☐ Porous
   ☐ non-porous
7. **Nature of the Surface 2**
   ☐ Smooth
   ☐ Rough
8. **Hand Washing Station Availability**
   ☐ Yes
   ☐ No
